# The Histone Deacetylase Inhibitory Potential of Chicken Egg Yolk Fat and Their Fatty Acid Composition

**DOI:** 10.1155/2023/6360487

**Published:** 2023-10-18

**Authors:** Meran Keshawa Ediriweera

**Affiliations:** Department of Biochemistry and Molecular Biology, Faculty of Medicine, University of Colombo, Colombo 08, Sri Lanka

## Abstract

Histone deacetylation is a key biochemical event associated with transcriptional regulation. Histone deacetylases (HDACs) mediate the deacetylation of histones. Fatty acids have been reported to function as histone deacetylase inhibitors (HDACi). The present instigation reports the HDAC inhibitory activity of egg yolks and egg yolk-derived fat of country and farm chicken for the first time. Egg yolks and fatty acids derived from both country (CCEF) and farm chicken (FCEF) demonstrated significant HDAC enzyme activity inhibition. Furthermore, egg yolks, CCEF, and FCEF exhibited DPPH free radical scavenging effects. The analysis of fatty acid profiles revealed varying degrees of saturated, mono-, and polyunsaturated fatty acids in the egg yolks. Palmitic acid (C16 : 0) was found to be the most abundant saturated fatty acid in both CCEF and FCEF. Among the monounsaturated fatty acids, oleic acid (C18 : 1) was the most abundant in both CCEF and FCEF. In terms of polyunsaturated fatty acids, a significant difference was observed in the content of linoleic acid (C18 : 2), an omega-6 fatty acid, and docosahexaenoic acid (C22 : 6), an omega-3 fatty acid, between CCEF and FCEF. These findings present exciting prospects for the development of histone deacetylase inhibitors based on egg yolk fat.

## 1. Introduction

Egg yolk is the yellow component of a chicken egg and is covered by the egg white or albumen. The yolk is a substantial source of proteins, triacylglycerol, phospholipids, fatty acids, cholesterol, and vitamins A, D, and E [1]. The leading countries in chicken egg production, such as China, the United States, India, Mexico, and Brazil, contribute to global egg production significantly (FAO, 2019) [2].

Country chickens, also known as village chickens, are raised in rural or village settings, setting them apart from the farm chicken breeds commonly found in urban areas and intensive poultry farming operations [3–7]. Country chickens mainly find their food by foraging, whereas farm chickens are fed formulated diets in an organized manner in controlled environments [5, 6]. Farm chicken eggs are easily accessible in local supermarkets and grocery stores, whereas country chicken eggs are typically sold at home scale in countries like Sri Lanka. Individuals often have their preferences for country and farm chicken eggs based on factors such as taste, price, availability, and cultural preferences. Scientific studies have revealed that the nutritional composition of country and commercial chicken egg yolks differs [8–13]. Notably, in Sri Lanka, people favor consuming country chicken eggs due to their perceived richer nutritional profile and more desirable taste [3].

The fatty acid profiles of egg yolks are distinct and characterized by a substantial presence of monounsaturated and polyunsaturated fatty acids [14, 15]. Among the monounsaturated fatty acids, oleic acid is the most predominant in the egg yolk, while palmitic acid stands out as the primary saturated fatty acid [14, 15]. Furthermore, egg yolks serve as an abundant source of long-chain omega-3 fatty acids, particularly eicosapentaenoic acid (EPA) and docosahexaenoic acid (DHA), both of which have been reported to offer various health advantages, such as reducing inflammation, enhancing cognitive function, and decreasing the likelihood of heart disease [1, 16, 17]. Linoleic acid (omega-6) and alpha-linolenic acid (omega-3) are also present in substantial quantities in egg yolks [18, 19]. Therefore, considering the fatty acid composition of egg yolks becomes essential for individuals targeting to improve their dietary choices in terms of wellness and overall health.

HDACs are a vital group of enzymes that contribute to transcriptional regulation [20–22]. Their key function involves the removal of acetyl groups from histone proteins, causing the condensation of chromatin structure. This condensation increases hindrance for transcription factors to access the DNA and initiate gene expression, resulting in transcriptional repression [20–22]. Furthermore, HDACs play essential roles in diverse biological processes, including cell differentiation, cell cycle progression, and DNA repair [20–22]. Altered HDAC function has been associated with numerous diseases, such as cancer, neurodegenerative disorders, and cardiovascular disease. Consequently, the development of HDAC inhibitors (HDACi) has emerged as a potential therapeutic approach for treating these conditions [20–22].

Short and long-chain fatty acids, hydroxamic acid derivatives, and benzamides have been identified as HDACi [20–22]. Several fatty acids such as butyric acid, valproic acid, caproic acid, docosahexaenoic acid (DHA), a DHA conjugate and eicosapentaenoic acid (EPA) and linoleic acid, herbs, food items, and gut bacteria which are rich in fatty acids exert HDAC inhibitory potential in cell-free and cell-based systems [22, 23]. Recently, a homologous series of odd-chain fatty acids (pentadecanoic acid (C15 : 0), undecanoic acid (C11 : 0), nonanoic acid (C9 : 0), heptanoic acid (C7 : 0), and, valeric acid (C5 : 0)) was identified as HDAC 6 inhibitors [24]. Considering the HDAC inhibitory potential of fatty acid and fatty acids-rich food items, it was hypothesized that egg yolk, a food item that is rich in fatty acids, could exert HDAC inhibitory potential. The hypothesis being tested in this investigation is whether egg yolk, which is known for its high fatty acid content, possesses HDAC inhibitory potential. To investigate this, egg yolk fat obtained from farm and country chicken eggs sourced from Sri Lanka was extracted and evaluated.

## 2. Methods

### 2.1. Chemicals and Organic Solvents

The chemical and organic solvents used in the present study were purchased from Sigma-Aldrich (St. Louis, MO, USA) unless otherwise stated. The total HDAC assay kit was purchased from Abcam, Massachusetts, USA (ab1432).

### 2.2. Collection of Eggs and Separation of Egg Yolk

**Figure 1:**
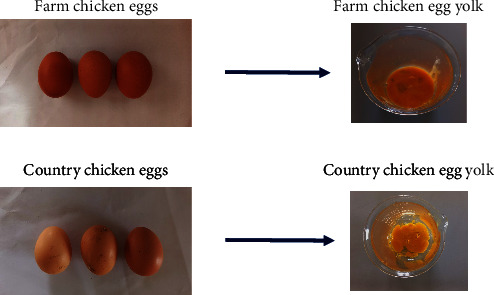
Farm chicken eggs, country chicken eggs, farm chicken egg yolk, and country chicken egg yolk.

Six farm and country chicken/village chicken eggs (3 each) were collected from a local market in Sri Lanka and a village farmer who sells eggs as a small scale business, respectively. The village farmer confirmed that the village chickens are fully foragers. Eggs were cracked, and the egg yolks were carefully removed into a beaker and subjected to fatty acid extractions (Figure 1). Except for the use of fatty acid extraction, small amounts of fresh/raw egg yolks were speed vacuumed to prepare stock solutions in ethanol and DMSO mixtures (8 : 1 v/v %) for DPPH radical scavenging and HDAC inhibitory assays.

### 2.3. Extraction of Fatty Acids

The Folch method was used to extract fats/fatty acids from farm and country chicken egg yolks with slight modifications [25]. The method involves adding chloroform and methanol to the sample, which results in the precipitation of lipids from the solution. The lipids are then separated from the solution by centrifugation and dried. Briefly, equal volumes of country and farm chicken egg yolks (approximately 4 mL) were mixed with 6 mL chloroform and methanol (2 : 1 v/v) and vortexed for 10–15 seconds in a glass vial. Then, ∼2 ml of distilled water was added to the glass vial, mixed well, and allowed to separate organic and aqueous layers overnight. The organic layer, which contains fat, was evaporated and submitted for fatty acid analysis (CCEF and FCEF separately).

### 2.4. Fatty Acid Analysis

The AOAC 996.06 method was used to analyze fatty acid compositions in the fat fractions obtained from farm and country chicken egg yolk [26]. The fatty acid compositions were expressed as relative peak area percentage (peak area relative to the total peak area %) according to standards established by the AOAC.

### 2.5. DPPH Assay

The DPPH free radical scavenging assay was performed to assess whether the egg yolks and egg yolk's derived fatty acid fractions possess DPPH radical scavenging ability. Briefly, a DPPH working solution (0.02 mM) was prepared in ethanol, and 180 *μ*L of the DPPH working solution was added to each well of a 96-well microplate. Then, 20 *μ*L of egg yolks dissolved in ethanol and DMSO mixture and egg yolk fat (CCEF and FCEF) dissolved in ethanol were added to each well, and the plates were incubated for 30 mins in a dark room. The final sample concentration was in the range of 5–40 mg/mL for egg yolk and fat samples. Following incubation, the absorbance of each well was recorded at 517 nm using a microplate reader. The percentage inhibition of the DPPH by the test samples was calculated using the following formula:

(1) $$\begin{array}{c} \%\text{Inhibition}=\left[\frac{\left(A-\text{control}-A-\text{sample}\right)}{A-\text{control}}\right]\times 100, \end{array}$$

where *A*-control is the absorbance of the DPPH solution without the sample and *A*-sample is the absorbance of the DPPH solution with the sample. The EC_50_ values were calculated using GraphPad Prism 5.0 software (GraphPad Software, Inc.)

### 2.6. HDAC Assay

The HDAC Activity Assay Kit (colorimetric) (ab1432) uses a colorimetric method to measure the activity of histone deacetylases (HDACs) according to the kit's protocol. The HDAC colorimetric substrate is a peptide that contains an acetylated lysine side chain. This peptide is incubated with a sample containing HDAC activity or inhibitory activity. If HDAC activity is present, the HDAC will deacetylate the lysine side chain of the peptide. This makes the peptide more susceptible to cleavage by an enzyme called the lysine developer. The lysine developer cleaves the peptide, releasing a chromophore. The levels of chromophore released is proportional to the amount of HDAC activity present in the sample tested. The absorbance of the chromophore was measured at 405 nm using a microplate reader. The final sample concentration was in the range of 1–4 mg/mL for egg yolk and egg yolk fat samples (CCEF and FCEF). Trichostatin A was used as the positive control.

### 2.7. Statistical Analysis

HDAC and DPPH free radical scavenging assays were carried out in triplicate (*n* = 3), and the results are expressed as the mean ± standard deviation (SD) of the three independent experiments. GraphPad Prism, version 5 (GraphPad Software, Inc.), software was used for statistical analysis. The Student's *t*-test was used to check whether there is a statistically significant difference in the fatty acid compositions between country chicken egg and farm chicken egg yolk fat. To analyze HDAC assay results statistically, one-way analysis of variance (ANOVA) was used with Dunnett's post hoc test for group comparisons (control vs. treated). *p* < 0.05 was used as the threshold for statistical significance.

## 3. Results

### 3.1. Fatty Acid Profiles of Egg Yolk Fat

**Figure 2:**
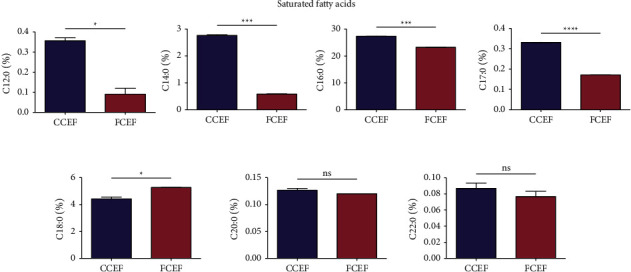
Saturated fatty acid compositions (%) of country chicken egg yolk fat (CCEF) and farm chicken egg yolk fat (FCEF) as analyzed by the AOAC 996.06 method. Lauric acid (C12 : 0), myristic acid (C14 : 0), palmitic acid (C16 : 0), margaric acid (C : 17), stearic acid (C18 : 0), arachidic acid (C : 20), and behenic acid (C : 22). The student's *t*-test was used to check whether there is a statistically significant difference in the fatty acid compositions between CCEF and FCEF. A *p* value of less than 0.05 between the two groups indicated a significant difference.

Figure 2 displays the saturated fatty acid compositions of fat extracted from country chicken (CCEF) and farm chicken egg (FCEF) yolks. Palmitic acid (C16 : 0) is the most abundant saturated fatty acid in both CCEF and FCEF. The CCEF contains higher levels of lauric acid (C12 : 0), myristic acid (C14 : 0), palmitic acid (C16 : 0), margaric acid or heptadecanoic acid (C17 : 0), arachidic acid (C20 : 0), and behenic acid (C22 : 0) compared to FCEF. Among these fatty acids, C12 : 0, C14 : 0, C16 : 0, and C17 : 0 showed a significant (*p* < 0.05) difference between the two groups. In contrast, stearic acid (C18 : 0) levels were found to be lower in CCEF compared to FCEF.

**Figure 3:**
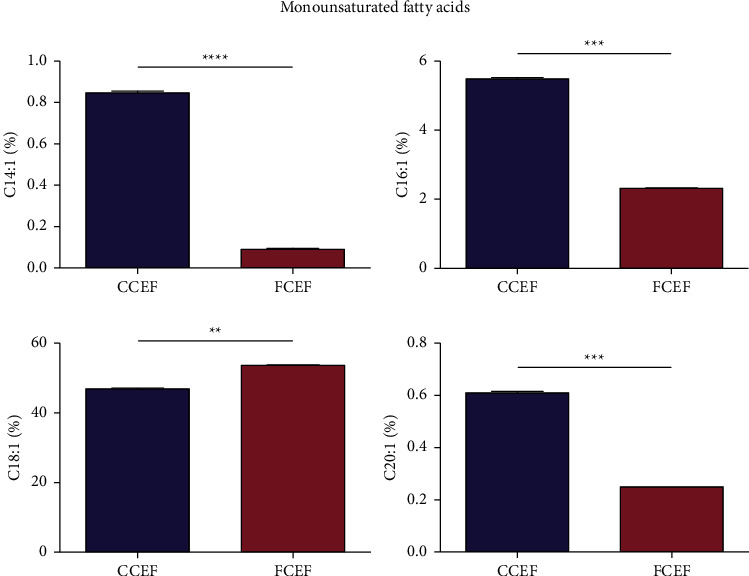
Monounsaturated fatty acid compositions (%) of country chicken egg yolk fat (CCEF) and farm chicken egg yolk fat (FCEF) as analyzed by the AOAC 996.06 method. Myroistoleic acid (C14 : 1), palmitoleic acid (C16 : 1), oleic acid (C18 : 1), and eicosenoic acid (C20 : 1). The Student's *t*-test was used to check whether there is a statistically significant difference in the fatty acid compositions between CCEF and FCEF. A *p* value of less than 0.05 between the two groups indicated a significant difference.

The concentration of monounsaturated fatty acids in CCEF and FCEF is shown in Figure 3. Among the monounsaturated fatty acids, oleic acid (C18 : 1) was the most abundant monounsaturated fatty acid in both the fat fractions obtained from CCEF and FCEF. FCEF displayed the highest C18 : 1 level compared to CCEF in a significant (*p* < 0.05) manner. Monounsaturated fatty acids such as myristoleic acid (C14 : 1), palmitoleic acid (C16 : 1), and eicosenoic acid (C20 : 1) were found in greater quantities in CCEF than in FCEF (Figure 3).

**Figure 4:**
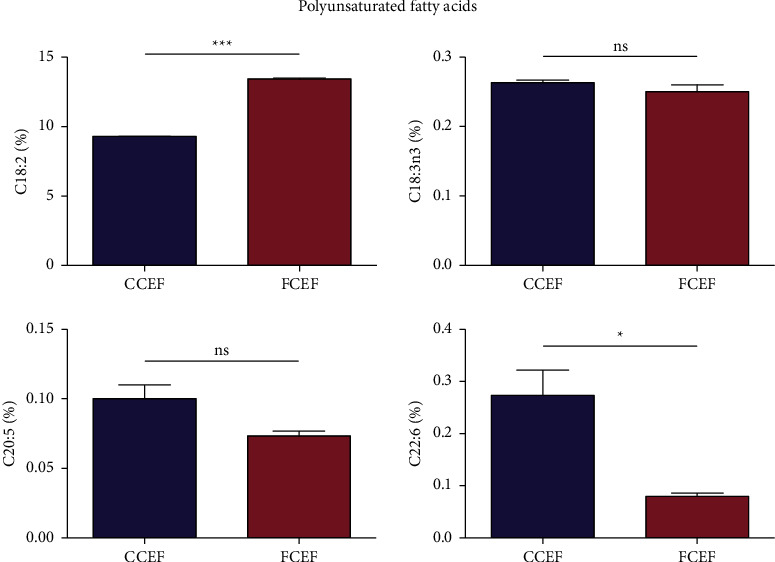
Polyunsaturated fatty acid compositions (%) of country chicken egg yolk fat (CCEF) and farm chicken egg yolk fat (FCEF) as analyzed by the AOAC 996.06 method. Linoleic acid (C18 : 2), *α*-linolenic acid (C18 : 3n3), eicosapentaenoic acid (C20 : 5), and docosahexaenoic acid (C22 : 6). The Student's *t*-test was used to check whether there is a statistically significant difference in the fatty acid compositions between CCEF and FCEF. A *p* value of less than 0.05 between the two groups indicated a significant difference.

As observed for the polyunsaturated fatty acid, linoleic acid (C18 : 2), an omega-6 fatty acid, and docosahexaenoic acid (C22 : 6), an omega-3 fatty acid, contents showed a significant difference between CCEF and FCEF. FCEF had higher C18 : 2 content than CCEF. On the other hand, CCEF showed a higher omega-3 fatty acid percentage (linolenic acid (C18 : 3n3), eicosapentaenoic acid (C20 : 5), and C22 : 6) compared to FCEF. However, CCEF had lower omega-6 fatty acid (C18 : 2) levels than FCEF (Figure 4).

**Figure 5:**
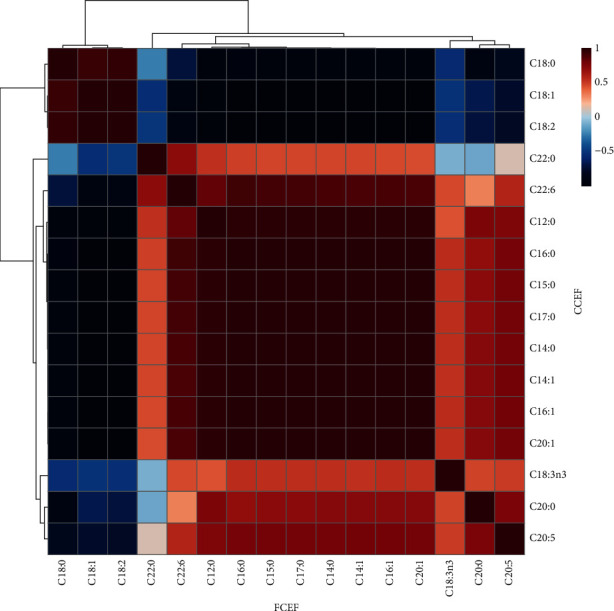
Pearson correlation heat map between country chicken egg yolk fat (CCEF) and farm chicken egg yolk fat (FCEF). The red color indicates a positive correlation, the blue color indicates a negative correlation, and the white/light blue color indicates no correlation. MetaboAnalyst 5.0 was used to generate the Pearson correlation heat map [27].

Figure 5 displays the heat map of Pearson correlation analysis among fatty acids and country chicken and farm chicken egg yolks. In the heat map, positive correlations are displayed in red, and negative correlations are displayed in blue. The darker the colors signify higher correlations between two groups.

### 3.2. Histone Deacetylase Inhibitory Effects of Fresh Egg Yolks and Egg Yolk Fat

**Figure 6:**
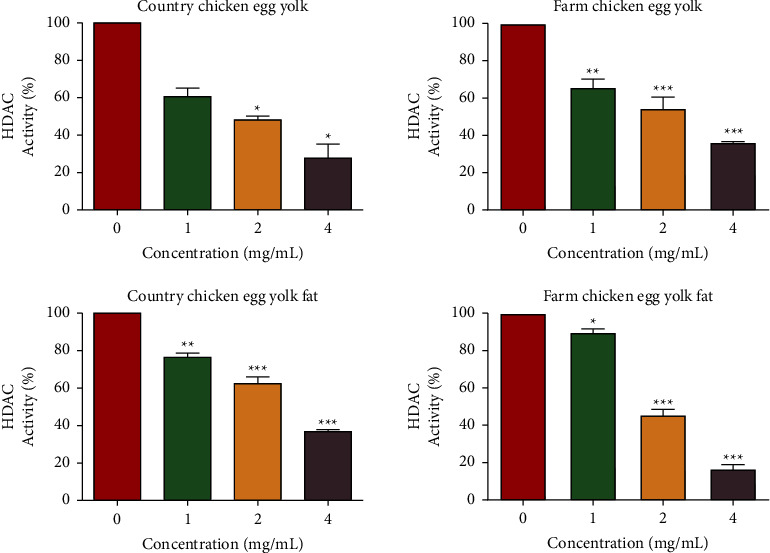
HDAC inhibitory potential of country chicken egg yolk, farm chicken egg yolk, country chicken egg yolk fat (CCEF), and farm chicken egg yolk fat (FCEF). One-way analysis of variance (ANOVA) was used with Dunnett's post hoc test for group comparisons (control vs. treated). *p* < 0.05 was used as the threshold for statistical significance.

Figure 6 illustrates the HDAC inhibitory potential of both fresh egg yolks and egg yolk fat. It is evident that both fresh egg yolks and egg yolk fat exhibit HDAC inhibitory potential in a dose-dependent manner when tested at concentrations ranging from 1 to 4 mg/mL. Notably, country chicken egg yolk displayed a higher HDAC inhibitory potential in comparison to fresh farm chicken egg yolk, as shown in Figure 6. At doses of 2 and 4 mg/mL, country chicken egg yolk displayed a reduction in HDAC enzyme activity, reaching 48.30% and 27.76%, respectively (Figure 6). Moreover, FCEF demonstrated a greater HDAC inhibitory potential than CCEF (Figure 6). The dosages of 2 and 4 mg/mL of FCEF reduced the HDAC enzyme activity to 45.70% and 16.75%, respectively. Trichostatin A, the positive control, exhibited potent HDAC inhibitory effects although these data are not displayed.

### 3.3. DPPH Radical Scavenging Effects

The EC_50_ values (concentration required to obtain a 50% antioxidant effect) of country and farm chicken egg yolks, CCEF, and FCEF are shown in Table 1. Fresh country chicken egg yolk and CCEF displayed the highest DPPH radical scavenging effects.

## 4. Discussion

The present investigation was planned to investigate the fatty acid composition, HDAC enzyme inhibitory, and DPPH radical scavenging potentials of country and farm chicken egg yolks found in Sri Lanka. The farm chicken industry in Sri Lanka is capable of meeting a considerable number of local requirements for chicken meat and eggs. The indigenous chicken production in Sri Lanka is an important component of the country's poultry industry. According to the available genetic diversity findings, Sri Lankan country chicken is closely related to the red and grey junglefowls [28].

The fatty acid composition of egg yolks can vary depending on the diet of the chicken that lays the egg. Eggs from country chickens, which are raised in free-range environments and have access to natural foods, tend to contain a higher proportion of polyunsaturated fatty acids (PUFAs) compared to eggs from farm-raised chickens fed a commercial diet [10, 29].

Oleic acid (C18 : 1) and palmitic acid (C16 : 0) were the most abundant monounsaturated and saturated fatty acids found in both CCEF and FCEF, respectively. Our results are in agreement with the previous investigations that have reported the presence of C18 : 1 and C16 : 0 in egg yolks [14, 15]. Fatty acids such as EPA and DHA have been extensively studied for their positive effects on heart, brain, eye, and mental health and inflammation and immune function [17, 30]. Country chick and farm chick egg yolks have been reported to contain higher omega-3 and omega-6 fatty acid contents, respectively [10, 11, 29]. The investigation by Lordelo et al., 2020 also demonstrated similar fatty acid profiles in country and farm chick egg yolks [10]. A study by Millet et al., 2006 demonstrated that the country chicken egg yolk had higher omega-3 fatty acids compared to a commercial breed [11]. Higher values of omega-3 fatty acids found in country chicken egg yolk compared to farm chicken eggs might be because country chickens grazed on fresh green plants. Green plants have been reported to contain a higher proportion of polyunsaturated fatty acids especially than commercial mixed diets [29, 31]. It is interesting to note that the results of the present investigation also demonstrated almost similar unsaturated fatty acid profiles in country and farm chicken egg yolks collected from Sri Lanka.

The *n* − 6/*n* − 3 ratio is a significant factor when it comes to maintaining good health. When the *n* − 6/*n* − 3 ratio is skewed towards an excessive intake of omega-6 fatty acids, it can lead to chronic inflammation, which is associated with numerous health issues such as cardiovascular disease, arthritis, and metabolic disorders [30]. It is interesting to note that the omega-6/omega-3 ratio of CCEF is lower than that of FCEF, which is a nutritionally significant indication [13].

The diet of chickens is strongly linked to the fatty acid composition of their eggs. The specific feed ingredients and composition can directly impact the fatty acid profile of chicken egg yolks. The presence of omega-6 fatty acids such as linoleic acid and omega-9 fatty acids such as oleic acid in diets can increase their concentration in the egg yolk. Ingredients such as corn, fish, canola soybean, and sunflower oil are rich sources of omega-6 fatty acids, which are important for normal growth and development [32, 33].

Egg yolks contain various antioxidants that can have beneficial effects on health. These antioxidants help protect against oxidative stress and reduce the damage caused by harmful free radicals in the body. One such antioxidant found in egg yolks is lutein, which is known for its role in promoting eye health and preventing age-related macular degeneration. Another important antioxidant present in egg yolks is zeaxanthin, which has been linked to reducing the risk of cataracts and improving overall vision. Moreover, egg yolks also contain vitamins A, E, and D, which have antioxidant properties and contribute to maintaining healthy skin, strengthening the immune system, and protecting against chronic diseases. In addition, egg yolk phospholipids and phosvitin have been reported to exert antioxidant effects [34–37]. According to the results of the present study, fresh country chicken egg yolk and CCEF demonstrated higher DPPH radical scavenging potential.

An investigation by Remanan and Wu, 2014 demonstrated that fresh egg yolk has a higher antioxidant capacity than fresh egg white [38]. Another investigation by Nimalaratne et al., 2011 reports the antioxidant effects of aromatic amino acids found in egg yolk [39]. Studies addressing the antioxidant effects of fresh egg yolks collected from country and farm chicken of Sri Lanka are extremely limited in the literature. Therefore, it is necessary to initiate novel investigations to assess the free radical scavenging effects of egg yolks obtained from different commercial and country chicken breeds in Sri Lanka.

Although some food items such as milk, graphs, and cruciferous vegetables have been reported as epigenetics diets [40], there is limited research on the effects of egg yolk fatty acids on HDAC (histone deacetylase) inhibition. HDAC inhibition refers to the ability to prevent or reduce the activity of these enzymes, which play a crucial role in the regulation of gene expression. Egg yolks are known to contain various fatty acids, including saturated, monounsaturated, and polyunsaturated fats. While some fatty acids have been shown to exert HDAC inhibitory effects, it is unclear which specific fatty acids in egg yolks may have this property. Fatty acids such as DHA and EPA have been reported to exert histone modifications [22]. Furthermore, the carbon side chain of fatty acid has been reported to play an important role in the inhibition of HDAC 6 activity [24]. As the carbon side chain of all fatty acids is structurally similar, it could be assumed that all fatty acids of egg yolk fat contribute to their HDAC inhibitory potential in varying degrees. However, to get a clear HDAC inhibitory profile of egg fat and to generate a correlation between individual fatty acids and HDAC inhibitory potential, it is necessary to investigate the HDAC inhibitory potential of each major component of egg yolks. Fatty acid profiling of egg yolks obtained from different countries and commercial chicken breeds found in Sri Lanka can provide valuable insights into the fatty acid composition of eggs.

## 5. Conclusion

This investigation demonstrates that egg yolks of country and farm chick eggs collected from Sri Lanka have distinct fatty acid profiles. Fresh egg yolks and fat isolated from country and farm chick eggs exert HDAC inhibitory effects and DPPH radical scavenging effects *in vitro*. This preliminary investigation provides a rational to investigate detailed fatty acid profiles, epigenetics effects, and antioxidant effects of different country and commercial chicken breeds available in Sri Lanka.

## Figures and Tables

**Table 1:** EC_50_ values (in mg/mL) obtained for fresh country and farm chicken egg yolks, CCEF, and FCEF from the DPPH free radical scavenging assay.

	EC_50_
Fresh country chick egg yolk	8.74 ± 2.03
Fresh farm chicken egg yolk	64.18 ± 9.48
CCEF	7.50 ± 3.50
FCEF	10.20 ± 10.22

## Data Availability

The datasets used and/or analyzed during the current study are available from the corresponding author upon reasonable request.
